# Supplementary material for: Complexome profiling of the Chlamydomonas psb28 mutant reveals TEF5 as an early PSII assembly factor
Source: Plant Cell. 2025 Mar 17;37(6):koaf055. doi: 10.1093/plcell/koaf055 (PMC12164590; doi:10.1093/plcell/koaf055)
Supplement: koaf055_Supplementary_Data [file koaf055_supplementary_data.zip › psb28_interactive.zip!/plots/intensity/qualityPlots/peptides/CCS5_Cre17.g702150.t1.2.html]

### Cre17.g702150.t1.2

Trivial name: CCS5  
SeqCoverage [median]: 23.60  
Mass: 26.44 kDa  
  
 

| Sequence | MissedCleavage | Mass [Da] | wtIntensity | muIntensity | Score |
| --- | --- | --- | --- | --- | --- |
| ASAAAAPDVMAGPSR | 0 | 1370.66 | 4514.45 | 14557.40 | 132.69 |
| ELVPQEYELEK | 0 | 1375.69 | 1968.61 | 9541.38 | 110.87 |
| TGEPQAAAVGR | 0 | 1055.54 | 388.91 | 2351.83 | 140.45 |
| VNFVMLNIENSK | 0 | 1406.72 | 444.10 | 5128.63 | 113.93 |
| WAPEAAEFGVR | 0 | 1231.60 | 0.00 | 1391.62 | 55.57 |
|  |  |  | 7316.1 | 32970.9 |  |
